# Microglial cells in astroglial cultures: a cautionary note

**DOI:** 10.1186/1742-2094-4-26

**Published:** 2007-10-15

**Authors:** Josep Saura

**Affiliations:** 1 Department of Cerebral Ischaemia and Neurodegeneration, Institute for Biomedical Research of Barcelona (IIBB), CSIC, IDIBAPS. 08036-Barcelona, Spain

## Abstract

Primary rodent astroglial-enriched cultures are the most popular model to study astroglial biology in vitro. From the original methods described in the 1970's a great number of minor modifications have been incorporated into these protocols by different laboratories. These protocols result in cultures in which the astrocyte is the predominant cell type, but astrocytes are never 100% of cells in these preparations. The aim of this review is to bring attention to the presence of microglia in astroglial cultures because, in my opinion, the proportion of and the role that microglial cells play in astroglial cultures are often underestimated. The main problem with ignoring microglia in these cultures is that relatively minor amounts of microglia can be responsible for effects observed on cultures in which the astrocyte is the most abundant cell type. If the relative contributions of astrocytes and microglia are not properly assessed an observed effect can be erroneously attributed to the astrocytes. In order to illustrate this point the case of NO production in activated astroglial-enriched cultures is examined. Lipopolysaccharide (LPS) induces nitric oxide (NO) production in astroglial-enriched cultures and this effect is very often attributed to astrocytes. However, a careful review of the published data suggests that LPS-induced NO production in rodent astroglial-enriched cultures is likely to be mainly microglial in origin. This review considers cell culture protocol factors that can affect the proportion of microglial cells in astroglial cultures, strategies to minimize the proportion of microglia in these cultures, and specific markers that allow the determination of such microglial proportions.

## Review

Our knowledge of the functional potentialities of astrocytes has not ceased to grow in the last decades. Astrocytes are now recognized as important players in fundamental CNS functions such as energy metabolism [1], neurotransmission [2], maintenance of the blood-brain barrier [3], extracellular ion homeostasis [4] or cerebrovascular regulation [5] and they also play key roles in neuroinflammation [6] or repair [7]. Much of this knowledge has been obtained through in vitro studies. Although astroglial cell lines exist, such as rat C6 or human U373, primary cultures are by far the most commonly used model to study astroglial biology in vitro. A note regarding terminology: Primary cultures are, strictly speaking, those prepared by plating cells directly after isolation from tissue. When a primary culture is subcultured we obtain secondary cultures, tertiary cultures, etc. In this review the term "primary culture" is used in a less strict way to include also subcultures, except in Additional File 1 in which "primary" means without subculture.

Primary astroglial-enriched cultures are easy to prepare, reproducible and versatile and can be obtained from virtually any CNS region. Most laboratories prepare astroglial-enriched cultures from mice and rats but human primary astroglial-enriched cultures are also common whereas other species are used more seldom. Astroglial-enriched primary cultures can be prepared from organisms of any age, including embryonic, foetal, neonatal, young, adult and old organisms. In terms of yield and purity, the optimal age is while astrogenesis peaks; this occurs after the peak of neurogenesis and before the peak of oligodendrogenesis. In mice and rats this optimal window spans a period from 2–3 days prenatal to 2–3 days postnatal for cortex and most CNS regions [8], but not for cerebellum where it spans between post-natal days 4 and 7 [9].

Most protocols for preparing astroglial-enriched cultures from rat/mouse late embryos/neonates are derived from the seminal work of Booher and Sensenbrenner [10] or the later modification by McCarthy and de Vellis [11]. A completely different approach, not discussed here, is the culture of acutely isolated astrocytes [12,13]. Astrocytes are able to grow in a great variety of in vitro conditions, however, and virtually every laboratory has created its own more or less modified protocol (see e.g. Additional File 1). In most of these protocols the dissected tissue is dissociated by mechanical and/or enzymatic digestion and the dissociated cells are plated. If adequate plating density, plate coating, medium composition and regime of medium changes are used, astrocytes proliferate rapidly and a confluent culture is obtained generally 7–14 days after plating. In these cultures type-I astrocytes form a monolayer with variable amounts of type II astrocytes. Since astrocytes constitute the most abundant cell type in these preparations, they are often described as astroglial, astroglial-rich, astroglial-enriched or even pure astroglial cultures. However, in none of these preparations do astrocytes represent 100% of cells. Depending on culture conditions, oligodendrocytes [11,14], neurons [15], various types of precursors [16,17], ependymal cells [14,17], fibroblasts [18-20], endothelial cells [20,17,21] or microglial cells [17,22] can be present in these cultures, generally in small proportions.

**Figure 1:**
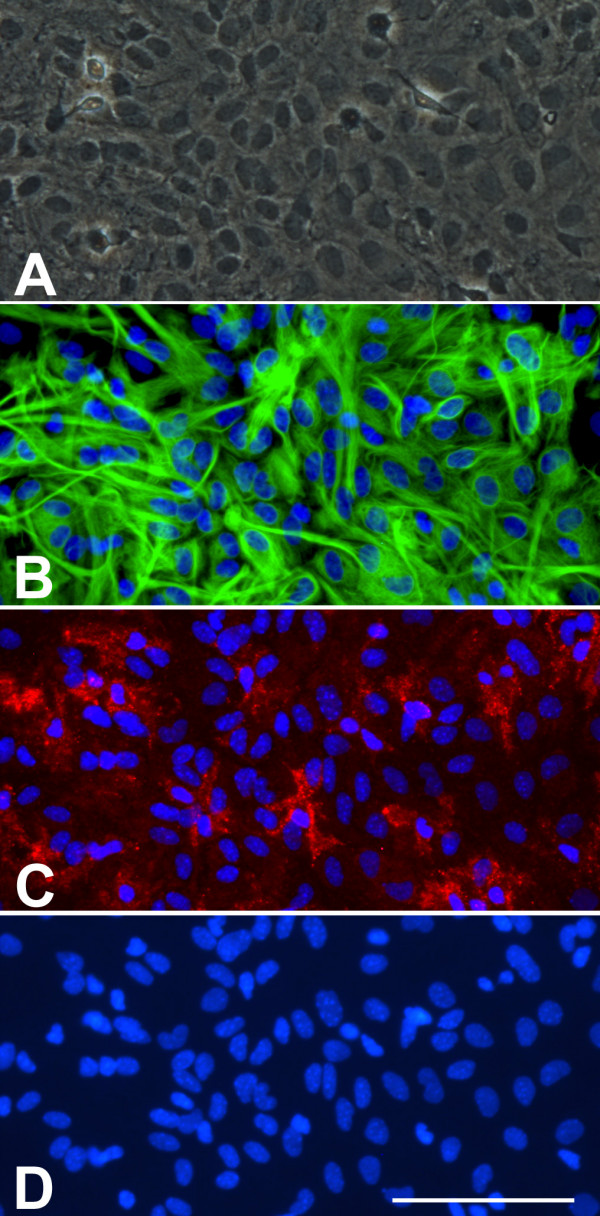
A) Phase contrast image of a confluent murine primary cortical mixed glial culture. A monolayer of type-I astrocytes is observed with some refringent microglia-looking cells. This image could suggest that this is an almost pure astroglial culture. B) GFAP immunostaining of the same field in A seems to confirm this impression. It is difficult to count how many astrocytes are in the field but since the whole field is covered by astrocytes one might conclude that indeed this is an almost pure astroglial culture. C) Immunostaining with the microglial marker CD11b reveals the presence of numerous (20) microglial cells in the field. These are mainly not the round refringent microglial cells typically recovered by shaking and found on top of the astrocytes. Instead these are more ramified cells, in direct contact with the bottom of the well, between the astrocytes or below them. D) Hoechst staining allows the easy quantification of the total number of cells in a culture. In this field there are 109 cells and microglial cells represent 18%. Hoechst 33258 staining also reveals the different nuclear morphology of astrocytes and microglia. Bar, 100 μm

The purpose of this review is to bring attention to the presence of microglia in astroglial cultures. In my opinion both the proportion and the role that microglial cells play in astroglial cultures are often underestimated. One possible explanation for such underestimation is historical. When the first protocols for astroglial cultures were established no good markers for microglial cells in vitro existed. As a result, the questions of whether microglial cells were present in astroglial cultures and in what proportion were not addressed in classical papers in the field [10,11,14]. These publications were used as a guide by many groups and this may partly explain why the presence of microglial cells remained unquestioned when good markers for microglial cells in vitro later became available. Another reason why microglial cells in astroglial cultures have sometimes been ignored is related to the image obtained after glial fibrillary acidic protein (GFAP) immunostaining, by far the most common approach to estimate the proportion of astrocytes in these cultures. Since astrocytes form a confluent monolayer, GFAP immunostaining results in an image in which virtually the entire cultured area is covered by astrocytes, giving the false impression that there is not much room for other cells (Fig 1). Authors may conclude that since there are no GFAP-free spaces, the proportion of non astroglial cells must be minimal and no further studies to establish the proportions of non astroglial cell types are deemed necessary. However, the reality is that non-astroglial cells can be present above and below the astroglial monolayer, and that specific markers should be used to estimate the proportions of these contaminating cell populations.

### Relevance of microglial cells in astroglial cultures

Why is it important to know the proportion of microglia in astroglial cultures and to keep this proportion as low as possible? First, because astrocytes and microglia are very different types of cells. One should not dismiss the presence of microglial cells in astroglial cultures with the thought that "after all, they are both glial cells". Their ontogenic origins are different – neuroectodermal for astrocytes, monocytic for microglia – their physiological roles are different, and many of their responses upon activation are also different. Microglia can do things that astrocytes cannot and small amounts of microglia can sometimes be responsible for the effects observed in cultures in which the astrocyte is the predominant cell type. If the relative contributions of astrocytes and microglia are not properly assessed one can erroneously attribute a microglia-derived effect to astrocytes, just because they are the predominant cell type. I will illustrate this point with the example of the production of NO in activated astroglial-enriched cultures.

### NO production by astrocytes and microglia in vitro

**Figure 2:**
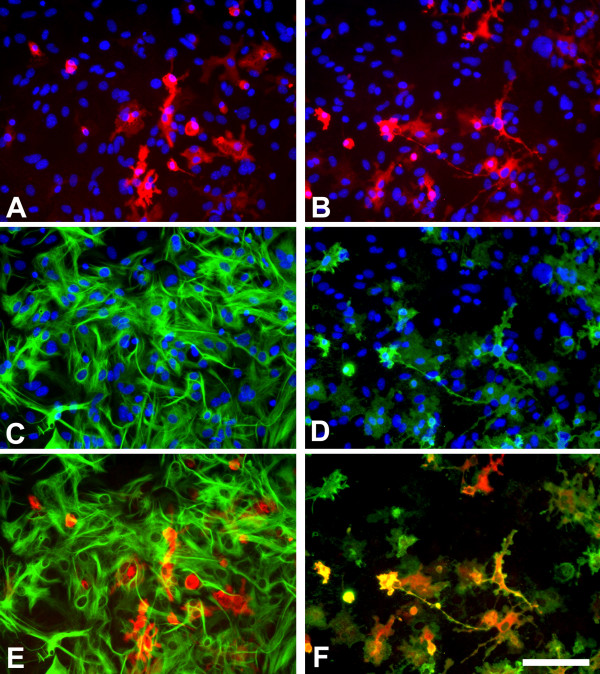
Murine primary cortical mixed glial culture were treated with LPS (1 μg/ml) and IFNγ (0.5 ng/ml) for 24 hours and immunostained for NOS2 (A, B), GFAP (C) or CD11b (D). A and C show the same field and E is their merged image. B and D show the same field and F is their merged image. In control cultures NOS2-immunoreactive cells were not observed (data not shown). There is (LPS + IFNγ)-induced NOS2 expression in numerous cells (A, B). NOS2-positive cells were almost never GFAP immunoreactive (A, C, E) indicating a lack of NOS2 expression in most astrocytes. In contrast, virtually all NOS2-positive cells (>98%) were identified as microglia by their CD11b immunoreactivity. Nuclei are counterstained with Hoechst 33258 in A-D. NOS2-positive cells were identified with a rabbit anti-NOS2 antibody (1:500, BD Biosciences), GFAP-positive cells with a mouse anti-GFAP antibody (1:1000, Sigma) and CD11b-positive cells with 5C6 mouse anti-CD11b antibody (1:400, Serotec). Bar, 100 μm.

A great amount of literature exists regarding NO production by astroglial-enriched cultures, particularly upon LPS activation of cultures. Most reports analyze NO production by measuring the accumulation of released NO in the culture medium and many assume that such NO production is astroglial. However, a review of the literature suggests that this assumption is not correct in many of these studies. NO can be produced by NO synthases 1, 2 and 3 (NOS1, NOS2, and NOS3, also known as cNOS, iNOS and eNOS, respectively) and these enzymes can be expressed by astrocytes [23]. The NOS2 inhibitor 1400W abolishes the LPS-induced NO production in rodent glial cultures [24] suggesting that in these experimental conditions NO production by NOS1 or NOS3 isoforms is negligible. Therefore, a sound approach to determine the cell type producing NO in LPS-activated primary glial cultures is to identify the NOS2 expressing cells. Table 1 summarizes all the studies that, to my knowledge, have addressed the question of what cell types express NOS2 in LPS-activated rodent astroglial-enriched or mixed glial cultures. It is clear from these data that in these cell preparations LPS induces NOS2 expression in microglia since co-localization of NOS2 with a microglial marker was observed in every study (n = 10) in which this was analyzed. The evidence that LPS also induces NOS2 expression in cultured rodent astrocytes is certainly weaker. In most studies (10 out of 16) the authors failed to observe any NOS2 positive astrocyte by NOS2-GFAP double labelling. Among the studies that have reported the presence of NOS2 positive astrocytes in LPS-treated rodent cultures, possibly the most convincing images are found in the recent reports of Hamby et al [25,26]. Interestingly, this group found that the number of NOS2-positive astrocytes is extremely low after LPS activation, and that this increases after activation with LPS + interferon-γ (IFNγ) and especially after activation with LPS + IFNγ + transforming growth factor β1 (TGFβ1) [25]. This indicates that the expression of NOS2 in activated astrocytes and microglia is dependent on the activation stimulus. Von Bernhardi et al [27] did show NOS2-positive astrocytes, but NOS2 expression was stronger in microglia. NOS2-positive astrocytes were also observed in the only study from this series performed on spinal cord astroglial cultures [28] suggesting a possible regional heterogeneity on the ability of activated astrocytes to express NOS2. Finally, some reports identified NOS2-positive cells as astrocytes by GFAP staining despite the fact that these cells had the morphology of microglia in vitro [29,30]. In my experience treatment of murine primary mixed glial cultures with LPS (1 μg/ml) and IFNγ (0.5 ng/ml) for 24 hours results in intracellular NOS2 immunoreactivity in numerous cells. Double labelling of NOS2 with GFAP or CD11b reveals that the great majority of NOS2 immunoreactive cells are microglia, as identified by CD11b immunoreactivity (Fig 2). Less than 2% of all NOS2 positive cells were CD11b-negative and less than 1% of all NOS2 positive cells were positive for GFAP.

**Table 1:** Astroglial and microglial NOS2 immunoreactivity in LPS-activated mouse/rat astroglial-enriched/mixed glial cultures. This table summarizes all the studies that, to my knowledge, have addressed the question of what cell types express NOS2 in LPS-activated rodent astroglial-enriched or mixed glial cultures. Studies using activating stimuli other than LPS or studies on human glial cultures are not included in this table. Reports describing the presence of NADPH diaphorase in LPS-treated astroglial-enriched cultures (e.g [77]) are also not included because NOS2 is only one of many brain enzymes that exhibit NADPH diaphorase activity [78].

**Ref**.	**Species-Age-Region**	**[LPS] μg/ml**	**Co-activators**	**Time post-LPS**	Presence in astrocytes*	**Presence in microglia (marker)**	**Notes**	Double labelling method**
[29] Fig 4	Mouse-Neonatal-Whole Brain	1	-	-	Yes (?)	Yes (Mac1)	"The number of GFAP/NOS2 positive cells was low". NOS2(+) cells have microglial morphology and not a clear GFAP staining	DAB-Ni and DAB
[68] Fig 2	Rat-Neonatal-Cortex	100	-	24 h	No	-	NOS2(+) cells have a microglial appearance but no double labelling is done	IF
[69] Fig 1	Rat-Neonatal-Cortex	1	-	24 h	No	Yes (Isolectin-B4)		IF
[70] Fig 1	Rat-Neonatal-Forebrain	1	-	12 h, 24 h, 48 h	No	-	NOS2(+) cells have a microglial appearance but no double labelling NOS2/microglia is done	DAB and AP
[71] Fig 3	Rat-Neonatal-Cortex	2	IFNγ (100 U/ml)	18 h	No	Yes (OX-42)		IF
[72] Fig 2	Rat-Neonatal-Cortex	2	IFNγ (100 U/ml)	18 h	No	Yes (OX-42)		IF
[73] Fig 3	Rat-Neonatal-Cortex	2	IFNγ (100 U/ml)	18 h	-	Yes (OX-42)	"All NOS2(+) cells were OX-42(+)"	IF
[74] Fig 2	Rat-Neonatal-Cortex	2	IFNγ (100 U/ml)	18 h	No	-		IF
[39] Figs 2,3,5	Rat-Neonatal-Cortex	10	-	48 h	No	Yes (OX-42)		IF
[30] Fig 3	Rat-Neonatal-Neopallium	0,025	IFNγ (100 U/ml)	24 h	Yes (?)	-	Some double GFAP-NOS2(+) cells do not look like astrocytes	IF
[75] Fig 5	Rat-Neonatal-Cortex	1	IFNg (100 U/ml)	48 h	No	Yes (Isolectin-B4)		IF
[28] Fig 1	Rat-Neonatal-Spinal cord	1	-	24 h	Yes	-	Double GFAP-NOS2(+) cells. Double NOS2-microglial staining not done because "these cultures had no OX42 positive cells"	IF
[76] Fig 3	Rat-Neonatal-Cortex	0,1	-	24 h	No	Yes (OX-42)	"All NOS2(+) cells were OX-42(+)"	IF
[27] Fig 2	Rat-Neonatal-Cortex	1	IFNγ (10 ng/ml)	14 h	Yes	Yes (Isolectin-B4)	"NOS2 staining is much stronger in microglia than in astrocytes"	IF
[24] Fig 6	Mouse-Neonatal-Cortex	1	CGS21680 (100 nM)	48 h	No	Yes (Tomato lectin)		IF
[26] Fig 6	Mouse-Neonatal-Cortex	2	IFNγ (3 ng/ml)	24 h	Yes	-	Double GFAP-NOS2(+) cells	IF
[25] Fig 8	Mouse-Neonatal-Cortex	2	IFNγ (3 ng/ml) and TGFβ1 (3 ng/ml)	14 h	Yes	-	Double GFAP-NOS2(+) cells. The number of NOS2(+) astrocytes is very low after LPS but high after TGFβ1 + LPS + IFNγ treatment	IF

* GFAP was the astroglial marker used in all the studies

** DAB: diaminobenzidine, DAB-Ni: DAB-Nickel; AP: Alkaline phosphatase; IF: immunofluorescence

Rodent astrocytes are certainly able to express NOS2 in vitro [31,32] and in vivo [23] and the production of NO by human astrocytes is also a confirmed observation [33,34]. Nevertheless, these results show that upon LPS or LPS + IFNγ activation of rodent astroglial-enriched/mixed glial cultures NOS2 is strongly expressed in microglial cells whereas the expression of NOS2 in astrocytes is weaker than in microglia if not completely absent. In agreement with this, the NO production induced by LPS in highly-enriched astroglial cultures, virtually devoid of microglia, is barely measurable by the Griess reaction whereas the same method reveals a robust NO production in microglial cultures or in mixed glial cultures [25,35-41]. These data suggest that LPS-induced NO production in rodent primary cortical astroglial cultures is mainly produced by "contaminating" microglial cells and not by astrocytes as often assumed.

There are similar concerns regarding studies identifying the cell of origin for apolipoprotein-E (apoE) production. In the CNS apoE appears to be produced in vivo by glial cells, mainly by astrocytes. Rodent cortical primary mixed glial cultures produce and release apoE [40,42-45]. Because of the in vivo data and because astrocytes are the main cell type in these cultures one might infer that apoE production in these cell preparations is basically astroglial in origin. However, highly-enriched astroglial cultures do not produce or release apoE whereas highly-enriched microglial cultures do [40], and apoE immunoreactivity in rodent mixed glial cultures co-localizes with microglial and not with astroglial markers [46]. Therefore apoE is another example showing that microglial cells, despite not being the most abundant cell type, can be responsible for observations made in astroglial-enriched/mixed glial cultures.

A great number of studies are published every year using astroglial-enriched cultures to describe the expression of all sorts of molecules, e.g. cytokines, chemokines, adhesion molecules. Most of these studies identify such expression by methods that lack cellular resolution (PCR, Western blot, ELISA, etc.) and many assume that the astrocyte is the cell type causing the observed effects. The examples of NOS2 and apoE show that this assumption may sometimes be incorrect, and indicate how important the estimation of the microglial proportion is when working with astroglial-enriched/mixed glial cultures.

### Microglial cells in astroglial cultures: how to estimate and affect their proportion

**Figure 3:**
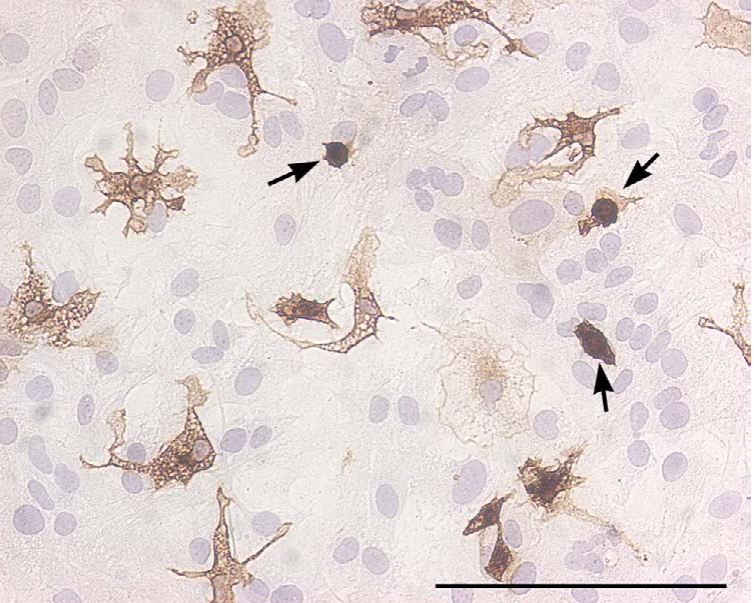
Round and ramified microglia in mixed glial cultures. Bright field image of a murine primary cortical mixed glial culture stained with the microglial marker Tomato lectin (brown) and counterstained with hematoxylin (blue). In this field the proportion of microglial cells is 13%. Three of them, identified with arrows, are round microglial cells with a strong lectin staining. These cells are easily identified by phase contrast by virtue of their refringency. In contrast, there are several microglial cells with ramified morphology and less intense lectin staining. These cells are non-refringent by phase contrast microscopy. Because of their weaker staining with various microglial markers and their non-refringency, the proportion of ramified microglial cells in astroglial-enriched/mixed glial cultures is often underestimated. Microglial cells were identified with biotin-labelled Tomato lectin (1:500, Sigma). Bar, 100 μm.

Determination of the proportion of microglia in astroglial cultures is seldom done (see e.g. Additional File 1), but in fact such determination is easy. Staining of microglia in fixed cultures with specific markers, antibodies or lectins, gives reliable results and, once stained, microglial cells are easy to count. In my experience, the antibodies ED1, which recognizes the lysosomal glycoprotein CD68, and OX-42, which recognizes CD11b, are good microglial markers in rat astroglial cultures [47,48], whereas the 5C6 anti CD11b antibody (see Fig 1) and the lectin from Lycopersicon sculentum (Tomato lectin) (see Fig 3) are good microglial markers in mouse astroglial cultures [24,49]. Other markers used to identify microglia in astroglial cultures include the antibodies F4/80 or anti-Ionized calcium binding adaptor molecule 1 (Iba1) and the lectins isolectin-B4, Griffonia simplicifolia agglutinin (GSA) or Ricinus Communis Agglutinin (RCA). Microglia can also be labelled with DiI labelled acetylated-Low Density Lipoprotein (DiI-Ac-LDL) which, unlike the microglial markers listed above, is used in living, unfixed cultures. Microglial cells, but not astrocytes, internalize and degrade Ac-LDL and the fluorescent probe DiI accumulates in intracellular membranes [22]. The protocol is rapid and simple. It is a good strategy to estimate the proportion of microglia in sentinel wells of multiwell plates that are subsequently used for experiments and also to follow changing proportions of microglia over time. A caveat of this method is that endothelial cells also take up Ac-LDL [50].

When establishing the proportion of microglial cells in astroglial cultures it is important to bear in mind that two populations of microglial cells exist in these cultures. On one hand, there are round refringent microglial cells, often named amoeboid, which are located on top of the astroglial monolayer and which can be recovered by shaking. On the other hand, there are nonrefringent, ramified microglial cells which are located below the astroglial monolayer or intermingled among the astrocytes and which can be isolated by mild trypsinization [51]. For many microglial markers the staining of refringent amoeboid microglial cells is stronger than that of ramified microglial cells and this may result in an underestimation of the proportion of ramified microglial cells (Fig 3). Similarly, the use of phase contrast microscopy to estimate the proportion of microglia in astroglial cultures will result in an underestimation because refringent amoeboid microglia are easily visualized with this approach but ramified microglial cells are often indistinguishable from the surrounding astrocytes [52]. An alternative approach to microscopy for estimating microglial proportions in astroglial-enriched cultures is flow cytometry, but this method so far has been seldom used [53].

What is the true proportion of microglia in rodent astroglial-enriched cultures? This may vary from less than 1% to 30% or even more. Several factors have an influence on this proportion. Here I will briefly review the effects of some parameters that may be important in this respect. For a review on methodological aspects of astroglial cultures please see [54].

#### Animal age

This is not a critical factor. There is no particular age that is especially suitable to obtain astroglial cultures with low proportions of microglia. It must considered that animal age does affects glial activation state, and therefore can affect cellular markers such as GFAP [55,56]. As was mentioned above, late embryos or newborn animals are generally preferred because they have the highest yields of both astrocytes and microglia vs. neurons.

#### Species

Rat cortical astroglial cultures that are virtually devoid of microglial cells can be obtained by subculturing at low density [40,39,57,18,58]. The same procedure has also been used in mouse cultures (see e.g. Additional File 1) but in my experience this procedure is less effective at reducing the microglial content in mouse than in rat cortical astroglial cultures.

#### Region

There are probably no major differences in the microglial content of astroglial cultures prepared from most CNS regions. An exception is the substantia nigra which is particularly rich in microglial cells. As a result, the proportion of microglial cells in mesencephalic mixed neuronal/glial cultures is 4 to 8 times higher than that in cortical or hippocampal cultures [59]. Therefore one would expect the microglial content in mesencephalic astroglial cultures to be also high (unless adequate strategies to minimize the microglial content are used).

#### Culture medium

DMEM is the most often used medium for astroglial cultures; MEM or DMEM:F12 are also common (see Additional File 1). Many variants of these basic formulations are used to prepare astroglial cultures, e.g. with high vs. low glucose, with or without HEPES, with or without glutamine or Glutamax, with or without sodium bicarbonate. A proper comparison on the suitability of these media combinations for astroglial and microglial culture has not been reported. From my experience astroglial cells grow well both in DMEM and DMEM:F12. However, microglial cells are less abundant in DMEM than in DMEM:F12 astroglial cultures (unpublished observations).

#### Medium changes

The normal metabolism of an astroglial culture causes a progressive reduction in nutrients in the culture medium. In nutritionally deprived astroglial cultures astrocytes do not survive more than a few days. In contrast, microglial cells not only do survive well but they rapidly proliferate [60]. Therefore, in order to keep a low microglial content in astroglial cultures it is important to change the medium often (every 2–3 days at least). Changing media less often, e.g. once a week, will result in astroglial cultures with higher microglial proportions.

#### Coating

Unlike neurons, astrocytes grow well on uncoated plastic or glass surfaces but, unfortunately, so do microglia. Most laboratories prepare their astroglial cultures on uncoated, polylysine- or polyornithine-coated plates. Based on the finding that laminin favors astroglial growth and inhibits microglial growth [61] we tested the effects of laminin-coating on astroglial cultures and found that indeed the microglial content in astroglial cultures was markedly reduced by laminin-coating [24]. Other groups have independently adopted the use of laminin-coating in their astroglial cultures [62,63]. In our laboratory, using uncoated plastic we can obtain highly-enriched astroglial cultures (<2% microglia) from rat, but not from mouse cortex. To prepare astroglial-enriched cultures from mouse cortex we routinely use laminin-coating.

#### Subculture

When a primary astroglial culture reaches confluence it can be trypsinized and subcultured. This procedure results in a secondary culture in which the proportion of microglia and other contaminating cells is reduced [11]. This simple step is very effective in rat astroglial cultures, especially if subculturing is done at relatively low densities (<50.000 cells/cm^2^). As mentioned before, it has also been used to prepare mouse astroglial cultures (see e.g. Additional File 1), although in my experience this procedure is less effective in this case.

#### Shaking

Shaking a confluent astroglial culture in an orbital shaker for 2 to 24 hours results in the detachment of many cells sitting on top of the astroglial monolayer, mainly microglia, type-II astrocytes and precursor cells [22]. Shaking is therefore an effective way to reduce the proportion of microglial cells in an astroglial culture. As noted before, not all microglial cells are on top of the astroglial monolayer. It is important to know that microglial cells located below or in between the astroglial monolayer will not be eliminated by shaking. Therefore, shaking can be used to prepare highly-enriched astroglial cultures provided the parent culture contains few microglial cells below or in between the astrocytes.

#### Cytosine arabinoside (Ara-C)

Ara-C is an antimitotic drug that is used alone or in combination with other strategies to reduce the presence of microglia and other contaminating cells in astroglial cultures. Ara-C must be added to cultures immediately after astrocytes have reached confluence. At this point astrocytes stop proliferating because of contact inhibition and microglia starts a phase of rapid proliferation. Most protocols use Ara-C at 5–10 μM and the treatment lasts 2–5 days.

#### Specific microglial toxins

Another strategy to reduce the microglial content of astroglial cultures is the use of drugs which are toxic to microglial cells and not to astrocytes. Although not used by most laboratories, this is an effective approach to keep the microglial content at its lowest especially when in combination with other strategies. L-leucine methyl ester, a lysosomotropic agent, has been often used to deplete microglia from astroglial cultures. First used in this respect by Giulian and Baker [22] at 5 mM for 2 hours, it has recently been re-evaluated and strong microglial depletion without astroglial "side-effects" has been obtained at 50–75 mM for 60–90 min [26]. Much less frequently used is clodronate, a bisphosphonate known to deplete cells of the monocyte lineage [64] that is used clinically in osteoporosis treatment. This drug markedly reduces the microglial content in hippocampal organotypic cultures [65] and could therefore be also useful in astroglial cultures, but to my knowledge it has not been tested in this respect.

#### Others

Strategies also exist aimed at increasing the purity of astroglial cultures by reducing the proportion of non-microglial contaminating cells, e.g. the replacement of glucose by sorbitol [14] which reduces the number of oligodendrocytes and ependymal cells, or the use of D-valine [18] in the culture medium which retards the growth of fibroblasts and meningeal cells. A detailed description of these methods lies outside the scope of this review which is focused on the presence and role of microglia in astroglial-enriched cultures. Finally, it is important to bear in mind that factors such as the source of serum or the type of plastic used in culture plates have probably an influence in the outcome of astroglial cultures and they could also affect microglial numbers.

In summary, microglial proportions can be determined in astroglial cultures and strategies exist that are useful at reducing these proportions.

## Conclusion

Several authors have noted previously that caution must be used when making a claim of pure astroglial cultures [22,26,52] and that attention must be paid to the presence of microglia in these cultures [66,67]. Although there are certainly groups using adequate methods to estimate and minimize the proportion of microglia, these claims have too often been ignored. In my opinion it is necessary that authors, referees and editors become aware of this question in order to reduce the number of publications in which the presence of microglia in astroglial-enriched cultures is ignored and especially those that attribute to astrocytes roles that are played by contaminating cells, particularly microglia. A few suggestions to achieve this goal are listed:

1. Microglial cells in astroglial-enriched cultures should always be identified by specific markers, should be counted, and the microglial proportion included in the paper together with the proportion of astrocytes and, if possible, with the proportions of other contaminating cell types.

2. Those interested in working with highly-enriched astroglial cultures should use an adequate protocol to reduce the proportion of microglia, e.g. laminin coating, shaking, subculture at low density, L-leucine methyl ester, frequent medium changes. However, the presence of microglia in astroglial cultures is in many cases desirable because it allows the astroglial-microglial cross talk that is extremely important in glial activation. The point is to be aware that microglial cells are present in these cultures and to be able to discriminate observed effects caused by astrocytes from those caused by microglia, from those caused by the combination of the two cell types, and from those caused by neither of them.

3. The term "pure astroglial cultures" is probably an oxymoron and it should be used as little as possible. I would suggest the term "highly-enriched astroglial cultures" for >99% astrocytes and the term "astroglial-enriched cultures" for >90% (or, ideally, >95%) astrocytes. When microglia are >10% the term "mixed glial cultures" is probably more appropriate.

4. High resolution techniques (e.g. immunocytochemistry, in situ hybridisation) should be used to clearly attribute an observed effect to a given cell type in astroglial cultures. The use of both highly-enriched astroglial and microglial cultures can also help in this respect. If these approaches are not possible the question should be left open and say, for example, "LPS induced an increase of X in astroglial-enriched cultures" which is a true observation instead of "LPS induced an increase of X in astrocytes" which could be a false deduction/misinterpretation.

## Abbreviations

apoE, apolipoprotein-E

Ara-C, Cytosine arabinoside

DiI-Ac-LDL, DiI labelled acetylated-Low Density Lipoprotein

GFAP, glial fibrillary acidic protein

GSA, Griffonia simplicifolia agglutinin

Iba1, Ionized calcium binding adaptor molecule 1

IFNγ, interferon-γ

LPS, Lipopolysaccharide

NOS2, NO synthase-2

RCA, Ricinus Communis Agglutinin

TGFβ1, transforming growth factor β1

## Competing interests

The author(s) declare that they have no competing interests.

## Supplementary Material

Additional file 1 Variability of the protocols for the preparation of astroglial-enriched cultures. This table shows methodological details of published protocols for preparing rodent astroglial cultures for >100 articles published in 2006. A majority of groups work with newborn rats/mice and use DMEM and 10% FBS, but the variations on the procedures used are large. Some aspects of the procedures such as the plating density, coating or frequency of medium changes are seldom found in the descriptions, making it difficult to compare data from different laboratories. Note also that microglial proportion estimates are infrequent. Click here for file
